# Biomimetic Wax Interfaces Facilitating Rehealable Polymer Composites

**DOI:** 10.3390/polym13183052

**Published:** 2021-09-09

**Authors:** Ching-Te Kuo, Chien-Chin Chen

**Affiliations:** 1 Department of Mechanical and Electro-Mechanical Engineering, National Sun Yat-sen University, Kaohsiung 804, Taiwan; 2 Department of Cosmetic Science, Chia Nan University of Pharmacy and Science, Tainan 717, Taiwan; hlmarkc@gmail.com; 3 Department of Pathology, Ditmanson Medical Foundation Chia-Yi Christian Hospital, Chiayi 600, Taiwan

**Keywords:** biomimetic, wax, rehealable, polymer composite

## Abstract

Epicuticular wax, the first protective film for numerous ground plant species, is crucial for modulating the evolution in plants. Since the waxy film is inherently thermoresponsive, many efforts focus on engineering materials for water/oil proofing, delivery, and collection, as well as microactuators by mimicking such film nature. Nonetheless, relatively fewer works address the mechanism of how the underlying substrates direct the reconstruction of waxy films while their temperature approaches the melting point. Here, we presented a strategy in which distinct frameworks of molten wax films could be examined among various substrates. Both “waxphobic” and “waxphilic” traits were first unveiled and could be achieved by the hydrophilic (water contact angle (WCA) = 42~82°) and hydrophobic (WCA = 109°) substrates, respectively. A theoretical model, based on experimental results, fluidic dynamics, and balance of surface energy, was developed to elucidate the above findings. Moreover, we demonstrated the above biomimetic epicuticular surface (BeSurface) can be applied for rewritable paper, erasable coding, and rehealable electronics without manual repairing. Remarkably, the healing time can be reduced down to 30 s, and the cycled folding test can be continued up to 500 times. All the new findings present the potentials of the BeSurface to improve the study of rehealable materials.

## 1. Introduction

Protective covering plays a crucial role in current electronic computing due to its adjustable capability that can meet the requirement for each individual user [1]. Likewise, epicuticular wax—the first line of protective covering in many ground plants—presents benefits to adapt and protect its holder against severe environmental pressures or natural enemies [2,3,4]. It can improve the reflectance of irradiation, alleviate the reduction of water loss, and restore limited water in drought areas. It has been demonstrated to be involved in the process of plant morphology and evolution. In addition, it is an inherently phase-changing material. Inspired by the mentioned characteristics, recent efforts have focused on engineering material structures that mimic the wax film for water/oil proofing, delivery, absorption, and collection [5,6,7,8,9]. Upon the hybridization of a wax and carbon nanotube composite, artificial muscle can be accomplished with a versatility of actuations [10,11,12]. In addition, glass surfaces micropatterned with a thin wax film (4.5 μm in thickness) can be further applied for cell patterning and drug screening, as well as in tumor spheroid cultivation [13,14]. All of these imply the versatile and controllable potential by adopting the nature of such wax films.

**Figure 1:**
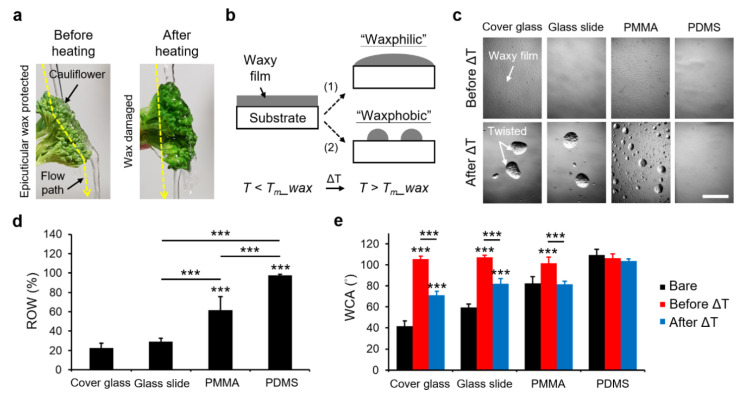
Reconstruction of molten wax films on different substrates. (**a**) Observation of the water flow paths through cauliflowers before and after heating. The heating condition was a boiling bath for 30 s. (**b**) Illustration showing the “waxphilic” and “waxphobic” phenomena at a temperature close to or higher than the melting point of wax. (**c**) Micrographs showing the derived architectures of molten wax films on cover glass, silane-coated glass slide, PMMA, and PDMS substrates, respectively. The heating condition was a hot plate at 65 °C for 30 s. The results after ΔT were acquired at room temperature. Scale bar = 500 μm. (**d**) Comparison of the ratio of wax area (ROW) carried on the substrates after ΔT. The substrate area for the examination was 1553 × 1125 μm^2^. (**e**) Comparison of water contact angle (WCA) on different substrates during no treatment (bare), and before and after heating conditions. Data represent the mean ± SD, *n* = 4 in (**d**) and *n* = 6 in (**e**). *** *p* < 0.001.

Leverage of temperature could impact the redeposition and reconstruction of epicuticular waxes in plant surfaces while subjected to global warming or heat treatment [15,16] (see also the cauliflower demonstration in Figure 1a). It may direct the evolution of plants or the long-term storage of fresh fruits. Since wax presents a phase transition at a temperature close to its melting point, a flow effect of the molten wax will dominate, and can be reversed according to the temperature transition. Based on this trait, recent works have engineered waxes for microfluidics and 3D-printing applications [17,18,19]. Furthermore, an artificial muscle was demonstrated with a programmable actuation using the heated tunability of wax [10]. Nonetheless, relatively few works have addressed the mechanism of how the reconstruction of wax films can be accomplished among different underlying substrates or determined the microscaled output of a wax film coated on a hydrophilic or a hydrophobic substrate.

For practical applications, rehealable electronics are attractive, with exciting progress in recent years [20,21,22,23,24]. It was proposed to be able to repair and restore damaged electrical functionality from several strategies, such as manual reassembly, external electronics for damage detection, bridging the damaged region by solvent or water, or self-healing by liquid–metal microdroplets. Nonetheless, challenges of time required for repairing still remain. For example, functional hydrogels were successfully used in wearable electronic devices with a healing period of 30 to 240 min [25,26]. Interestingly, M.A. Darabi et al. presented a newly conductive self-healing (CSH) hydrogel to advance the repairing time down to 1 min [27]. Although the repairing time was improved, the overall procedure was not user-friendly; instead, one was required to manually align and connect the damaged regions with additional efforts. This may discourage the practical usage of such a repairing technique, for example, for foldable smart phones and foldable screens. In addition, the time requirement would be increased up to several days if no additional auxiliary was applied [25].

To address the above issues, we present a strategy in which a thin wax film is coated on the examined substrate (Figure 1b). The film, which we called the biomimetic epicuticular surface (BeSurface), mainly consists of a mixture of hydrocarbon molecules containing 20~40 carbon atoms. Its melting point (*T*m_wax) is approximately 46 to 68 °C. We hypothesized two distinct characteristics of reconstructing the films could be categorized due to the phase transition of wax at *T* > *T*m_wax, termed as “waxphilic” and “waxphobic”. Since the activity of molten film strongly depends on the surface property of underlying substrates, the relevant applications for rehealable materials using BeSurface will be discussed in the following sections.

## 2. Materials and Methods

### 2.1. Coating with Waxy Film on Examined Substrates

The coating of the waxy film on the substrates was modified according to our previous work [13]. First, uniform pressure was applied to a sandwiched set of a foil sheet, a thermoresponsive sheet (Parafilm “M”), and a plain PDMS sheet (10: 1 *w*/*w* base to cure, Sylgard 184) at 70 °C for 30 s. Afterwards, the PDMS sheet, which had absorbed liquid-phase paraffin wax, was peeled off and immediately stamped onto the examined substrates for another 30 s at room temperature. The substrates examined in this work included a cover glass (Deckglaser; WCA = 42°), a silane-coated glass slide (511614, Muto Pure Chemical CO., LTD; WCA = 60°), PMMA (WCA = 82°), and PDMS (WCA = 109°). After peeling off the PDMS stamper, the waxy film coated on substrates was achieved with a thickness of approximately 1 μm.

### 2.2. Laser Direct Writing on Waxy-Film-Coated Substrates

The activity of laser writing on the examined substrates was performed by a CO_2_ laser engraver (Nova-35, Thunderlaser; 30 W). The maximum energy density of the laser was 3250 J/mm^2^. Parameters adopted for patterning circular shapes were set as “drill hole” modes, and the drilling time was set from 0.0005 to 0.3 s with a 4% power and a 10 mm/s moving velocity. As a result, the corresponding energy densities applied were 0.065 to 39 J/mm^2^. For patterning with line shapes, parameters were set as “cutting” modes, and the moving velocities were set from 5 to 70 mm/s with a 4% power and an identical 10 mm length. The energy densities were then found to be 260 to 18.6 J/mm^2^ accordingly.

### 2.3. Preparation of Heat-Healable Metal–Elastomer Composite Electrode (HHCelectrode)

The waxy film was first coated on the PDMS substrate according to the above approach. Then, 100 μL of silver flake solution (327077, Sigma; diluted in 70% ethanol with a 4:1 *w/w*) was then dispensed to cover the surface of the wax-coated PDMS. Following heating at 70 °C for 3 min, the entire set was covered with a thin sheet of PDMS as the protective layer.

### 2.4. Statistical Analysis

A one-way TukeyHSD ANOVA test was used to compare data from more than two groups; *p* < 0.05 was considered to be statistically significant.

## 3. Results and Discussion

### 3.1. Locomotion of Thermoresponsive Films on Underlying Substrates

We examined the reconstructed wax films on different substrates, including a cover glass, silane-coated glass slide, poly (methyl methacrylate) (PMMA) and polydimethylsiloxane (PDMS) at 65 °C for 30 s (Figure 1c). Interestingly, the reconstructed film resulted in separately twisted wax architectures on both glass substrates and many wax satellites from PMMA. In contrast, no difference was detected in the regular films using PDMS before and after heating (Figure 1c,d).

Upon the examination of the water contact angle (WCA), moreover, no significant difference was presented by PDMS between the bare conditions and the conditions before and after heating, as compared to other substrates (Figure 1e and Figure S1; please see the Supplementary materials for additional information). For the glass and PMMA substrates, the results indicated that the molten wax film would show a contracted trait to form twisted microstructures (Figure 1c). Furthermore, these wax-coated substrates (glass and PMMA) restored the wettability to water (Figure 1e). In other words, it could partially elucidate the differentiation shown in Figure 1a—two different water flow paths through cauliflower before and after heating.

### 3.2. Theoretical Model for the Locomotion of Thermoresponsive Films

**Figure 2:**
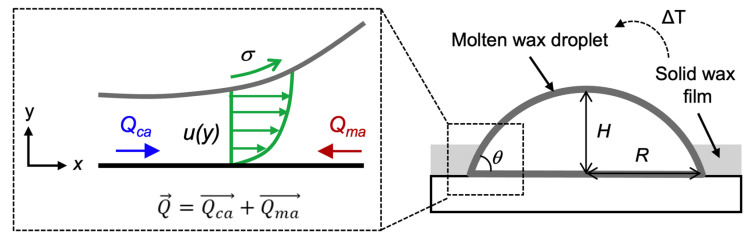
Theoretical modeling of the phase-transition activity of the waxy film, where *H*, *R*, *θ*, *Q_ma_*, and *Q_ca_* denote the height, radius, and contact angle of wax droplet; and the flow rates induced from Marangoni and capillary effects, respectively. *σ* is force applied along with the fluidic flow, and *u*(*y*) is the flow velocity.

Figure 2 illustrates the theoretical model, in which *H*, *R*, *θ*, *Q_ma_*, and *Q_ca_* denote the height, radius, and contact angle of the wax droplet; and flow rates induced from Marangoni and capillary effects, respectively. Since the induced flow from the molten wax was very sticky and the Reynolds number (Re) was assumed to be quite small (Re << 1), we assumed this flow activity could be described following the creeping flow:

(1) $$\nabla \vec{P}=\mu \nabla^{2}\vec{U}$$

where *P* and *U* denote the pressure applied on the flow and the velocity of the flow, respectively; and *μ* is the dynamic viscosity of the molten wax. Upon the assumption that the fluid layer was thin, the free surface was nearly parallel to the substrate, no friction was considered, and the substrate was in a nonslip condition, the governing Equation (1) could be approximated as:

(2) $$\frac{dP}{dx}=\mu \frac{d^{2}u}{dy^{2}}$$

where *u* is the velocity, varied only with the y axis. The boundary conditions were *u* = 0 at *y* = 0 and *u* = (*dσ*/*dx*)/*μ* at *y* = *h*, where *σ* is the surface force applied in the fluidic flow and *h* is the height of the fluid. The velocity *u* was therefore determined as:

(3) $$u=\frac{y^{2}}{2\mu }\frac{dP}{dx}+\frac{y}{\mu }\left(\frac{d\sigma }{dx}-h\frac{dP}{dx}\right)$$

and the net flow rate *Q* could be acquired as:

(4) $$Q=\overset{h}{\underset{0}{\int }}udy=\frac{h^{2}}{2\mu }\frac{d\sigma }{dx}-\frac{h^{3}}{3\mu }\frac{dP}{dx}$$

The pressure *P* acting in the fluidic flow could be simplified as *P* = *ρg*(*h*−*y*) − *σ*(*d*^2^*h*/*dx*^2^), where *ρ* and *g* are the density of molten wax and the gravity, respectively. Since the fluidic layer was assumed to be very thin, the pressure *P* could be then expressed as *P* = −*σ*(*d*^2^*h*/*dx*^2^), and the derivative *P* versus the *x* axis could be expressed as:

(5) $$\frac{dP}{dx}=-\left(\frac{d\sigma }{dx}\frac{d^{2}h}{dx^{2}}+\sigma \frac{d^{3}h}{dx^{3}}\right)$$

Applying Equation (5) with Equation (4), we can therefore modify the net flow rate *Q* as:

(6) $$Q=\frac{h^{2}}{2\mu }\frac{d\sigma }{dx}+\frac{h^{3}}{3\mu }\left(\frac{d\sigma }{dx}\frac{d^{2}h}{dx^{2}}+\sigma \frac{d^{3}h}{dx^{3}}\right)$$

According to the lubrication approximation, Equation (6) can be re-expressed as [28,29]:

(7) $$Q=\frac{h^{2}}{2\mu }\nabla \sigma +\frac{h^{3}}{3\mu }\nabla \left(\sigma \nabla^{2}h\right)$$

where the first term of Equation (7) is similar to the Marangoni flow induced from the heating on the wax droplet, mainly based on the surface tension gradient. The second term in Equation (7) refers to the Laplace capillary flow induced from the surface tension transition, mainly based on the wax contact angle. Upon the assumption that the fluidic layer is very thin, we can simplify Equations (6) and (7) as:

(8) $$\begin{array}{c} Q=Q_{ma}+Q_{ca}=\frac{H^{2}}{2\mu R}∆\sigma +\frac{H^{4}}{3\mu R^{3}}\left(\sigma +∆\sigma \right)\\ Q_{ma}=\frac{H^{2}}{2\mu R}∆\sigma \\ Q_{ca}=\frac{H^{4}}{3\mu R^{3}}\left(\sigma +∆\sigma \right) \end{array}$$

Considering the balance of the molten wax droplet on the substrate, the adhesive force to the substrate (i.e., the normal term of surface force *σ*) must be equal to the normal term of the Marangoni force Δ*σ*. Here, we assumed that the weight of such a droplet is very small, and therefore neglected the effect of gravity. Besides, the net capillary force *σ_sa_* (the sum of *σ_la_* and *σ_ls_*) must equalize the horizontal Marangoni force, where *σ_sa_*, *σ_la_*, and *σ_ls_* represent the surface forces from substrate to air, liquid to air, and liquid to substrate interactions, respectively. The *σ_la_* could be assumed to be as *σ*. This indicates that the horizontal *σ* would be equal to or smaller than the horizontal Δ*σ*. Therefore, the balance of wax droplet activity should satisfy both the two conditions as listed below:

(9) $$\begin{array}{c} ⊥:\;\left|\sigma \sin\theta \right|=\left|\frac{H}{\sqrt{R^{2+}H^{2}}}∆\sigma \right|\Rightarrow R_{\sigma ⊥}=\left|\frac{\sigma }{∆\sigma }\right|=\left|\frac{1}{\sin\theta \sqrt{1+{\left(\frac{1}{\Psi }\right)}^{2}}}\right|\\ ∥:\;\left|\sigma \cos\theta \right|\leq \left|\frac{R}{\sqrt{R^{2+}H^{2}}}∆\sigma \right|\Rightarrow R_{\sigma ∥}=\left|\frac{\sigma }{∆\sigma }\right|\leq \left|\frac{1}{\cos\theta \sqrt{1+\Psi^{2}}}\right| \end{array}$$

where we neglected the directional effect and considered the absolute value instead. *Ψ* is the ratio of height *H* to radius *R* of the wax droplet, and can be expressed as [14]

(10) $$\Psi =\frac{H}{R}=\frac{1-\cos\theta }{\sin\theta }$$

**Figure 3:**
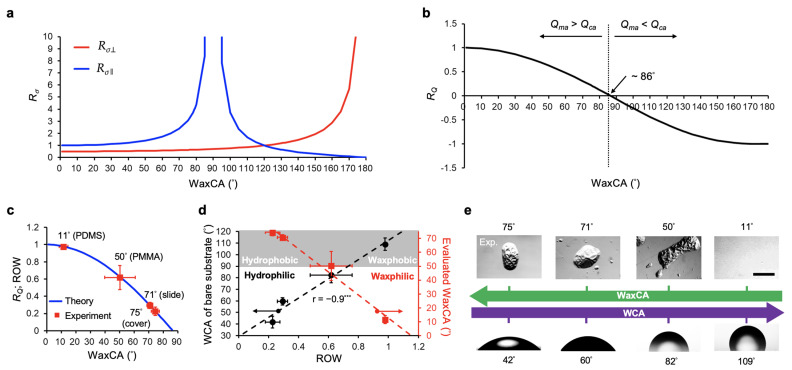
Theoretical characteristics of the phase transition of waxy films on underlying substrates. (**a**) Profiles *R_σ_*_⊥_ and *R_σ_*_‖_ versus WaxCAs from 0° to 180°. (**b**) Profile of *R*_Q_ versus WaxCAs from 0° to 180°. The critical WaxCA was 86° when *Q*_ma_ = *Q*_ca_. (**c**) Theoretical trend between the ratio of competed flow rate (*R*_Q_ ≡ ROW) and the wax contact angle (WaxCA). The WaxCA of substrates was evaluated by fitting the measured ROWs accordingly. (**d**) Relationship between the WCA or the WaxCA and the ROW on different substrates after heating the coated wax film at 65 °C for 30 s. (**e**) Summary of the trend between WCA and WaxCA derived from different substrates. Scale bar = 200 μm. Data represent the mean ± SD, *n* = 4. Pearson’s r = −0.9 (*** *p* < 0.001) indicated a significantly reversed trend between WCA and WaxCA in (**e**).

The trend of the force ratio *R_σ_* is shown in Figure 3a.

To further model the reconstruction of the molten wax films as observed in Figure 1c, we introduced a factor *R_Q_*, the ratio of competing flow rates from Marangoni and capillary flows, which is defined as:

(11) $$R_{Q}=\frac{\left|Q_{ma}\right|-\left|Q_{ca}\right|}{\left|Q_{ma}\right|+\left|Q_{ca}\right|}=\frac{1.5-\left|\Psi^{2}\left(1+R_{\sigma ⊥}\right)\right|}{1.5+\left|\Psi^{2}\left(1+R_{\sigma ⊥}\right)\right|}$$

where the profile of *R_Q_* is shown in Figure 3b, and the transition angle from positive to negative *R_Q_* values was approximately 86°. The ratio of wax area (ROW) indicated the percentage of reconstructed wax areas on the substrate after heating. Since the aspect ratio of waxy film is relatively small (thickness/width = 6 × 10^−4^), this ROW could supposedly correspond to the ratio *R*_Q_, in which |*Q_ma_*| − |*Q_ca_*| resembles the final area of the molten film and |*Q_ma_*| + |*Q_ca_*| resembles the total area of substrate. Upon the assumption of conservation of mass and neglecting the evaporation of molten wax in this model, we could therefore correspond the ratio *R*_Q_ (Equation (11)) to the ROW as ROW ≡ *R_Q_*.

To evaluate the WaxCA of the examined substrates, we applied the ROW measured as shown in Figure 1d to fit the ratio *R*_Q_ from Equation (11). The evaluated WaxCAs against the cover glass (WCA = 42°), glass slide (WCA = 60°), PMMA (WCA = 82°), and PDMS (WCA = 109°) were 75°, 71°, 50°, and 11°, respectively (Figure 3c). Interestingly, the characteristics of the WaxCA and WCA versus the ROW had an opposite linear relationship (Figure 3d). This showed that, for hydrophilic substrates (i.e., WCA = 42°, 60°, and 82°), they acquired WaxCAs of 75°, 71°, and 50°, respectively. In addition, the hydrophobic PDMS substrate acquired a “waxphilic” property, with a WaxCA of 11°.

The factor *R_Q_* (Equation (11)) seemed to dominate the resulting pattern of the heated wax film. The *R_Q_* of PDMS was 0.98, with a very smooth wax pattern after heating (Figure 1c), which was 1.58, 3.38, and 4.45 times more than the PMMA (*R_Q_* = 0.62), glass slide (*R_Q_* = 0.29), and cover glass (*R_Q_* = 0.22), respectively. The ratios of Marangoni to capillary flows were evaluated as 99, 4.26, 1.79, and 1.56 for the PDMS, PMMA, glass slide, and cover glass, respectively. Lower *R_Q_* values for both the glass substrates contributed to a larger capillary flow to Marangoni flow ratio, as compared with others. It therefore led to the separately twisted wax microstructures and some wax satellites after heating, as shown in Figure 1c. In contrast, the largest *R_Q_* from the PDMS substrate contributed to the smooth wax cover after heating, due to its larger Marangoni flow to capillary flow ratio. For the PMMA substrate, it preserved a moderate *R_Q_* value and a ratio of Marangoni flow to capillary flow 2.54 times more than both the glass substrates. This suggested that, after heating, the PMMA substrate also contributed the twisted wax microstructures, but formed more wax satellites than the glass substrates.

These above results summarize a new finding showing the surface properties of underlying substrates dominate the locomotive transition of thermoresponsive films (Figure 3e). They demonstrated that a substrate with a higher WCA will acquire a lower WaxCA, and vice versa. The hydrophobic PDMS substrate (WCA = 109°) possessed a “waxphilic” property; in contrast, both the hydrophilic cover glass (WCA = 42°) and silane-coated glass slide (WCA = 60°) were “waxphobic”. A moderate property was detected in the PMMA substrate (WCA = 82°).

### 3.3. Demonstration of Rehealability of BeSurface by Laser Direct Writing

**Figure 4:**
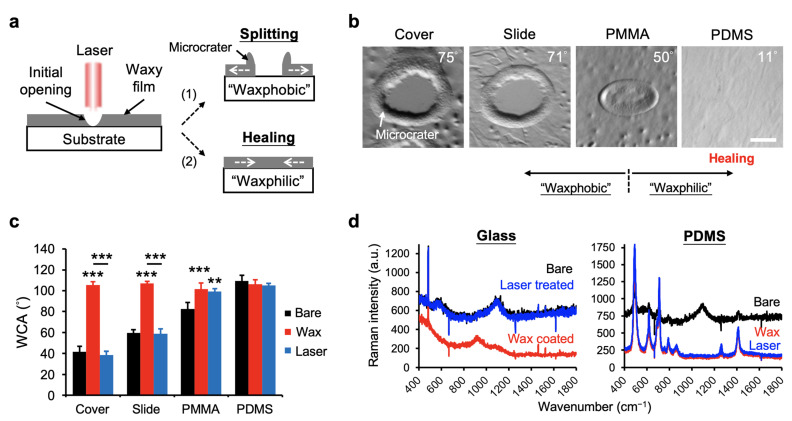
Laser direct writing on waxy films coated on different substrates. (**a**) Illustration showing two waxy patterns carried by laser writing—splitting and healing based on “waxphobic” and “waxphilic” substrates, respectively. (**b**) Micrographs showing the representative wax patterns formed by a 0.13 J/mm^2^ CO_2_ laser on different substrates. Scale bar = 50 μm. (**c**) Comparison of WCA on different substrates during no treatment (bare), and treatments with a waxy film coating (Wax) and laser writing (Laser). (**d**) Raman spectra of the cover glass and PDMS substrates during the three different treatments. Data represent the mean ± SD, *n* = 4~6. ** *p* < 0.01 and *** *p* < 0.001.

To realize the rehealability of the BeSurface at the microscale, we employed a CO_2_ laser (Nova-35, Thunderlaser, New Taipei City, Taiwan; 30W) acting on the wax films coated on the examined substrates. The schematic of the working principle is illustrated in Figure 4a, in which two differential patterns were assumed based on the surface properties of molten wax on the substrate. With a specific laser energy applied, an opening on the wax film was achieved for the “waxphobic” substrate. In contrast, healing the initial opening was detected from the “waxphilic” substrate. Upon applying a 0.13 J/mm^2^ dot laser energy, a circular pattern was achieved for both the cover glass and silane-coated glass slide, while only a small opening and no opening were detected for the PMMA and PDMS substrates, respectively (Figure 4b). These corresponded to the assumption illustrated in Figure 4a, since PDMS was supposedly “waxphilic”, but glass is “waxphobic” (Figure 3e). To further compare the patterning efficiency, we compared the WCA and the surface material composition using Raman spectra (iHR550 Micro-Raman spectrometer, HORIBA, Taipei, Taiwan) from different substrates, with and without laser treatments (Figure 4c,d). The results showed that the WCAs measured from glass substrates presented a significant difference between the wax-coated and the laser-treated surfaces, whereas no significant difference was detected for the PDMS substrate. A significant removal of the wax film was detected only for the glass substrates, indicating glass or “waxphobic” material could be effectively adopted for further applications of laser patterning. Remarkably, the “waxphilic” BeSurface (e.g., PDMS substrate) had rehealable potential, because it rearranged and restored its damaged wax film after heating. The details of its potential applications will be discussed later.

### 3.4. Rewritable Composite Paper Using BeSurface

**Figure 5:**
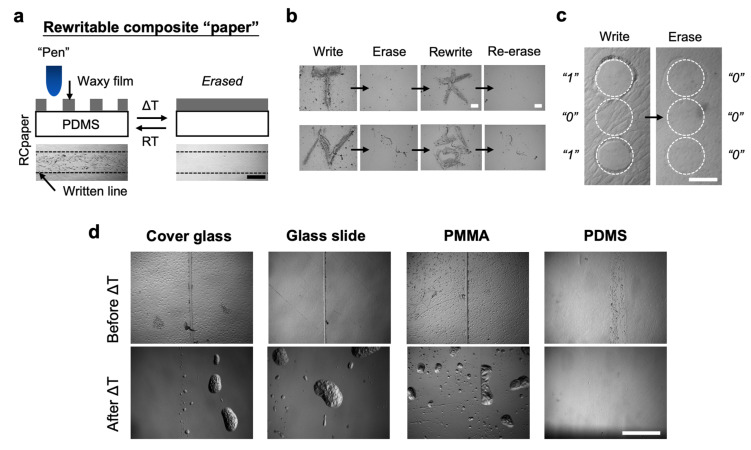
Rewritable composite paper (RCpaper). (**a**) Concept of the RCpaper with a rewritable ability. (**b**) Micrographs showing the repeated write and erase activities performed on the waxy film-elastomer composite paper. Each row panel indicates an identical location when writing or erasing. The writing activity was accomplished manually with tweezers. The erasing was completed at 65 °C for 30 s. (**c**) Demonstration of the erasable coding completed in the RCpaper. The dot pattern was performed with a 0.33 J/mm^2^ CO_2_ laser. The symbol of “1” indicates the dot region without covering with waxy film. In contrast, the region covered or re-covered (by heating) with wax is denoted as “0”. The example shows the “1 0 1” code could be erased or reset to be “0 0 0”. (**d**) Micrographs showing the comparison of rewritable traits of the four substrates used. The ΔT indicates that the RCpaper was heated at 65 °C for 30 s. Scale bars are 200 μm in (**a**), 500 μm in (**b**), 100 μm in (**c**), and 500 μm in (**d**).

Figure 5a shows the working principle of the rewritable composite paper using BeSurface. A waxy film of 1 μm thickness was coated on the PDMS elastomer, which was termed as the RCpaper. Since the PDMS was supposedly “waxphilic”, cracks induced from external forces were recovered or erased when the RCpaper was heated at a temperature close to the melting point of wax. Figure 5b demonstrates the rewritable ability of the RCpaper. First, two English characters were applied manually with tweezers on the determined locations. Second, the RCpaper was heated at 65 °C for 30 s and observed at room temperature. No residual patterns or cracks were detected. Third, two Chinese characters were reapplied at the identical locations. Finally, the observation was made following the erasing procedure, and the re-erasing activity was detected. Figure 5c demonstrates the typical coding and erasing activity by using the laser patterning technique described above. A larger laser energy of 0.33 J/mm^2^ was applied to generate an unhealable pattern on the RCpaper. The patterned codes were finally rehealed and erased by simply heating for 30 s. Moreover, the PDMS BeSurface behaved with a rehealable optimization compared with other substrates tested, e.g., glass and PMMA (Figure 5d).

### 3.5. Rehealable Composite Electrode Using BeSurface

**Figure 6:**
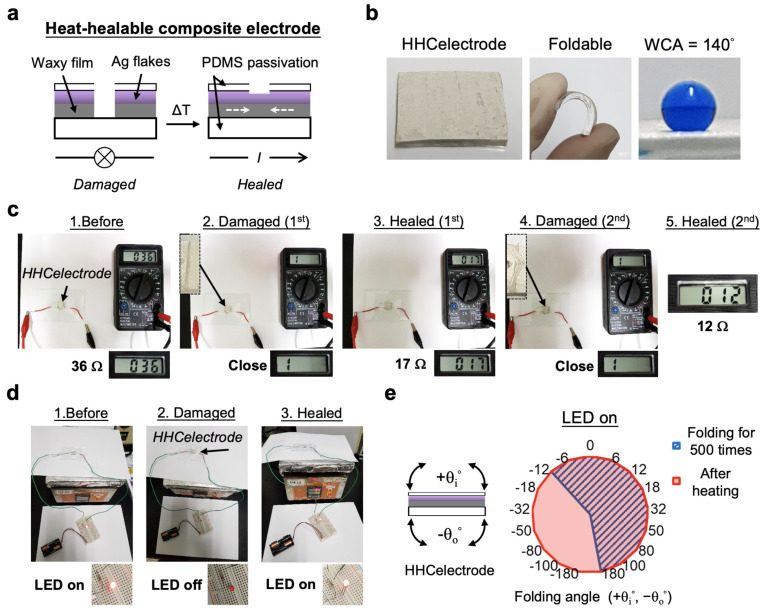
Heat-healable metal–elastomer composite electrode (HHCelectrode). (**a**) Concept and design of the HHCelectrode. “*I*” denotes the open circuit when the damaged electrode was rescued. (**b**) Characteristics of the HHCelectrode. (**c**) Photographs showing the sequenced cracking and healing processes completed with the HHCelectrode, by measuring the resistance of the electrode. The size of the electrode was 1.24 × 1.24 cm^2^. The cracking activity was accomplished by cutting with tweezers. The size of the line crack was approximately 0.8~1.2 mm. The healing was completed at 60 °C for 1 min. (**d**) Demonstration of the rehealable ability of the electrode by lighting a LED. The electrode, with an area of 1.3 × 1.3 cm^2^, was in series both with a 510 Ω resistor and an LED triggered by two 1.5 V batteries. Damaging and healing were completed by cutting with tweezers and heating at 60 °C for 1 min, respectively. Panel 3 shows the continuous observation of the healed electrode after 1.5 h. (**e**) Characteristics of the foldable HHCelectrode by lighting a LED. Each measurement of individual folding angles was performed for 500 folds. The electrode had an area of 2.3 × 1.2 cm^2^. All measurements of the healed electrodes were performed at room temperature.

To demonstrate the feasibility for rehealable applications, we incorporated the merits of the “waxphilic” PDMS and silver microflakes to fabricate the termed heat-healable wax–metal–elastomer composite electrode (HHC electrode), as illustrated in Figure 6a. The circuit was damaged when a crack was presented. Upon heating close to *T*_m_wax_, a liquidus wax film carrying electrically conductive micro-flakes dominated to bridge the crack, and thus the circuit was assumed to be rescued. Figure 6b shows the typical properties of the fabricated HHC electrode, including the appearance of the electrode, the foldable activity, and the hydrophobic merit with a 140° WCA. We first demonstrated the heat-healable activity of the electrode by applying cracks on it and simultaneously measuring its resistant transitions during the healing process (Figure 6c). Before damaging, the initial resistance of the electrode was measured as 36 Ω. After the first damage and healing process, the resistance was lowered to 17 Ω. The heat healing was then performed at 60 °C for 1 min. Following the second damage and healing process, a smaller resistance of 12 Ω was acquired. This suggested that the reconstruction of the wax–metal composite presented a more compact assembly with neighboring silver flakes to increase the electrical conduction. A further demonstration of the heat-healable ability was also successfully performed by connecting the electrode with an LED (Figure 6d; see also Video S1 in the supplementary materials for additional information). In addition, the HHCelectrode countered the folding tests up to 500 times per test, in which the inner folding (+*θ*_i_) sustained LED lighting from 0° to 180°, and the outer folding (−*θ*_o_) from 0° to −12° (Figure 6e). After healing, the circuit damaged by outer folding (−12° to −180°) could be totally rescued *θ*.

Rehealable materials have been demonstrated with an outstanding contribution in recent years [20,21,22,23,24]. In this paper, alternatively, we introduced another approach, and the RCpaper and the HHCelectrode could also achieve the above demand using the BeSurface technique ( a and a). This new concept allowed the rehealing of damaged material surface without the need for manual reassembly or external equipment for locating the damage. Compared to other works using manual and auxiliary efforts for healing damages [25,27], our labor-free approach could indeed improve the rehealable function more efficiently. Although the proof of conceptual results showed a limited outer folding activity, it did not lessen the capability and potential for the applications of rehealable electronics (Figure 6e). Remarkably, the times needed for repairing the RCpaper and HHCelectrode were as quick as 30 s and 60 s, respectively (Figure 5 and Figure 6). Less than 60 s was a relatively superior improvement of time compared to 240 min using functional hydrogel healing [26]. In addition, such a time improvement was comparable to the CSH hydrogel technique, [27] which requires 1 min for healing. These results indicated that the BeSurface technique possesses potentials for improving the study of rehealable materials. In future studies, the improvements relevant to the folding and extending activities of the electrode need to be further considered.

## 4. Conclusions

We have presented a biomimetic epicuticular rehealable surface (BeSurface) technology, in which a new mechanism termed as “waxphobic” and “waxphilic” transition was first unveiled. The BeSurface, a thin thermoresponsive wax film coated on a substrate, was inspired by the epicuticular wax on plant surfaces. The locomotion of the molten film highly depended on the surface properties of the underlying substrates. PDMS, with a 109° WCA, was determined to be a “waxphilic” substrate, based on the prediction of the theoretical model developed in this paper. In contrast, glass substrates were categorized as “waxphobic” materials. Upon discovering the merits of the BeSurface, we further applied it to emerging the applications of rewritable wax paper, coding and erasing, and rehealable electronics. These applications demonstrated that the repairing time could be reduced to 30 s. Altogether, our study opens a new avenue targeting the locomotive transition of thermoresponsive films, and is anticipated to ignite innovative applications, such as surface engineering on wearable and rehealable electronics, as well as emerging smart materials.

## Data Availability

The data presented in this paper are available upon request from the corresponding authors.

## Supplementary Materials

The following are available online at https://www.mdpi.com/article/10.3390/polym13183052/s1, Figure S1: Altered WCA between the before- and after-heating treatments on the waxy-film-coated substrates; Video S1: Demonstration of the rehealable ability of the HHCelectrode by lighting an LED.
