# Supplementary material for: Biomimetic Wax Interfaces Facilitating Rehealable Polymer Composites
Source: Polymers (Basel). 2021 Sep 9;13(18):3052. doi: 10.3390/polym13183052 (PMC8467712; doi:10.3390/polym13183052)
Supplement: Supplementary file 1 [file polymers-13-03052-s001.zip › Supplementary information.pdf]

Supplementary Information for

Biomimetic Wax Interfaces Facilitating Rehealable Polymer Composites

Ching-Te Kuo[1*,**] and Chien-Chin Chen[2,3**]

[1]Department of Mechanical and Electro-Mechanical Engineering,
National Sun Yat-sen University, Kaohsiung, Taiwan, R.O.C.
[2]Department of Cosmetic Science, Chia Nan University of Pharmacy and Science, Tainan 717, Taiwan, R.O.C.
[3]Department of Pathology, Ditmanson Medical Foundation Chia-Yi Christian Hospital, Chiayi 600, Taiwan, R.O.C

[*]Prof. Ching-Te Kuo. E-mail: chingtekuo@mail.nsysu.edu.tw
[**]These authors contributed equally to this work.

Supplementary information includes one figure and one video.

Supplementary Figure

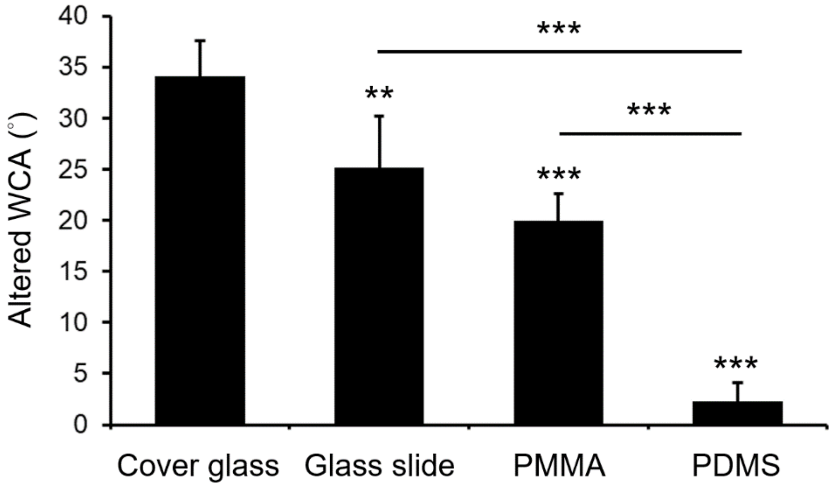

Figure S1. Altered WCA between the before and the after heating treatments on waxy film-coated substrates. High level of altered WCA indicates a severe damage of waxy film coated on substrate. It turns out the PDMS is of affinity with molten waxes more than the other substrates, suggesting PDMS could be categorized as a “waxphilic” material. The heating treatment is at 65 ˚C for 30 s.

Supplementary Video

Video S1. Demonstration of the rehealable ability of the HHCelectrode by lighting a LED. The electrode is placed on a hot plate. The crack is performed by manually cutting with a tweezer. The heating process from room temperature (25 ˚C) to the designed temperature (around 60 ˚C) will take 150 seconds. Meantime, the LED starts to be lightened transiently. It suggests the solid waxy film transforms into a fluid phase and initializes to bridge the cracked area at that time. After 1 min (at the time 210 s), the LED steadily lightens. It indicates the time needed for re-healing the cracked electrode is approximately 1 min when the crack is around 0.8 ~ 1.2 mm.
